# Parenchymal central nervous system involvement in aggressive B-cell lymphoma: retrospective analysis of clinical and MRI features in a Chinese population

**DOI:** 10.1186/s12883-019-1511-3

**Published:** 2019-11-04

**Authors:** Yuchen Wu, Yaming Wang, Xuefei Sun, Xueyan Bai, Jun Qian, Hong Zhu, Qu Cui, Ruixian Xing, Yuedan Chen, Qing Liu, Jiayuan Guo, Nan Ji, Shengjun Sun, Yuanbo Liu

**Affiliations:** 1 Department of Hematology, Beijing Tiantan Hospital, Capital Medical University, Nan Si Huan Xi Lu 119, Fengtai District, Beijing, 100070 China; 2 Department of Neurosurgery, Navy General Hospital of PLA, Beijing, China; 3 Department of Neurosurgery, Beijing Tiantan Hospital, Capital Medical University, Beijing, China; 4 Neuroimaging Center, Beijing Tiantan Hospital, Capital Medical University, Beijing, China

**Keywords:** Brain, Parenchymal lesions, Magnetic resonance, Lymphoma, Central nervous system

## Abstract

**Background:**

Secondary central nervous system lymphoma (SCNSL) is defined as secondary central nervous system (CNS) involvement in patients with systemic lymphoma. It is considered a profoundly adverse complication with inferior clinical outcome. Parenchymal involvement in the CNS in aggressive B-cell lymphoma is not frequently seen and remains a diagnostic dilemma.

**Methods:**

In our study, we retrospectively analyzed the clinical and magnetic resonance imaging (MRI) features of 26 parenchymal SCNSL patients. In addition, we compared MRI features of SCNSL and primary CNS lymphoma (PCNSL) patients after 1:1 propensity score matching. Also we presented two SCNSL cases with atypical MRI appearance.

**Results:**

Among SCNSL patients, the median CNS relapse time was 3 months, and multiple lesions were found in 76.9% of the cases. In PCNSL, this percentage was 42.3% (*p* = 0.011). None of the SCNSL patients and 23.1% of the PCNSL patients had solitary infratentorial lesions (*p* = 0.003).

**Conclusions:**

The majority of parenchymal involvement occurred within the first year of systemic lymphoma, in which mostly cases presenting with multiple and supratentorial locations, unlike what was found in PCNSL.

## Background

CNS lymphoma (CNSL) is an aggressive brain neoplasm that can involve the brain, meninges, spinal cord, and eyes. Secondary CNS lymphoma is defined as secondary CNS involvement in patients with systemic lymphoma [1]. Diffuse large B-cell lymphomas (DLBCLs) are the most common lymphoid neoplasms in adults, in which they account for approximately 32.5% of NHLs diagnosed annually. Secondary CNS involvement, which affects approximately 5% of patients with DLBCL, is considered a profoundly adverse complication with a median post-SCNSL overall survival of only 3.9–7.2 months [2, 3].

SCNSL can be generally divided into three conditions: systemic lymphoma combined with CNS involvement at presentation (combined disease), CNS involvement at the time of systemic relapse or progression (CNS with disease progression) and isolated CNS relapse despite systemic remission (isolated CNS disease) [4, 5]. The patterns of CNS involvement can be categorized as leptomeningeal, parenchymal, eye or combined. Spinal cord, peripheral nerve, or systemic involvement is uncommon as an initial manifestation of CNS lymphoma [6]. Despite intrathecal injection and intravenous application of methotrexate for CNS prophylaxis, 5% of systematic DLBCL patients eventually present with involvement in the central nervous system [7].

A diagnosis of SCNSL is usually made based on a combination of clinical presentation, radiological manifestations (enhanced MRI), and cerebral spinal fluid tests (conventional cytology and flow cytometry) [1]. Enhanced MRI of parenchymal SCNSL in symptomatic patients was highly informative [8]. However, patients with systematic DLBCL usually received corticosteroids included chemotherapy, and imaging features and differential diagnostic considerations may be altered by exposure to corticosteroids or in a setting involving immunosuppression [1]. Those patients may share similar symptoms and brain MRI characteristics with other neurologic disorders, including primary brain tumor, demyelinating disease, autoimmune or paraneoplastic syndromes, or CNS infection according to previous report [9]. SCNSL is challenging to detect, especially in the early stage, due to its diversity of magnetic resonance imaging (MRI) patterns and the complicated immune status of patients [10, 11]. Parenchymal SCNSL and PCNSL share some common MRI manifestations but their response to treatment and prognosis were distinct [12–14]. No current report describes the clinical and MRI features of parenchymal involvement of aggressive B-cell lymphoma in the Chinese population. In this study, we aim to summarize the unique presentation of parenchymal CNS involvement in DLBCL by comparing with primary central nervous system lymphoma on conventional MRI, to help early differential diagnosis and early detection of this fatal disease.

## Methods

### Patients

Clinical data were retrospectively reviewed at the Department of Hematology, Beijing Tiantan Hospital, Capital Medical University (Beijing, China) and the Department of Neurosurgery, Navy General Hospital (Beijing, China) between 2012 and 2019. There were a total of 26 SCNSL (19 from Navy General Hospital and 7 from Beijing Tiantan Hospital) and 26 PCNSL patients (all from Beijing Tiantan Hospital). All of them were HIV-negative. The present study protocol was approved by the Ethics Committees of Beijing Tiantan Hospital and Navy General Hospital. All patients gave written informed consent to participate in this study.

### Diagnosis of CNS lymphoma

The diagnosis of CNS relapse was based on the combination of clinical CNS features, radiological findings and histological findings of tumors. All PCNSL patients had histologically confirmed, 24 (92.3%) received stereotactic biopsy and 2 (7.7%) underwent intracranial tumor resection. Among SCNSL patients, 24 (92.3%) received stereotactic biopsy or intracranial tumor resection, and 2 (7.7%) were diagnosed by enhanced MRI. The immune-histochemical markers CD20, CD10, BCL-6, BCL-2, MUM1, CD138, EBER and Ki-67 were analyzed and viewed by an experienced hematopathologist, who categorized them by the Han’s algorithm.

### Imaging

Contrast enhancement MRI data were complete in all patients. All scans were evaluated by two experienced neuroradiologists regarding their number, location, T1 and T2 signal characteristics, patterns of contrast enhancement, diffusion properties. The location of the masses was classified as cerebral white matter, deep gray matter, brainstem and cerebellum, and further as supratentorial and/or infratentorial.

### Statistical analysis

The distributions of the characteristics of the patients were examined using the χ2 test. All statistical analyses were performed using SPSS 17.0 (SPSS, Inc., Chicago, IL, USA). *P* < 0.05 was considered to indicate a statistically significant difference.

## Results

### Characteristics of SCNSL patients at initial systemic disease diagnosis

Clinical findings are shown in Table 1**.** Half of the SCNSL patients (*n* = 13) were older than 60 years old when diagnosed with systematic aggressive B cell lymphoma. Extranodal involvement was observed in 14 (53.8%) patients, breast involvement in 15.4% (*n* = 4), testicular involvement in 11.5% (*n* = 3), and involvement of the intestines, parotid gland, oral cavity, rhino, orbit and spleen in 26.9% (*n* = 7). The histological findings were DLBCL in 92.3% (*n* = 24) of the patients, mantle-cell lymphoma in 3.8% (*n* = 1), and follicular lymphoma in 3.8% (*n* = 1). For the initial treatment prior to CNS involvement, 22 patients with isolated CNS disease received chemotherapy prior to CNS disease. 42.3% (*n* = 11) of the patients used Rituximab-containing therapy. Only 7.7% (*n* = 2) of the patients received intravenous HD-MTX for CNS prophylaxis. As for 4 SCNSL patients with combined disease, two patients had breast and CNS involvement, the other two had bone marrow and CNS involvement. They did not receive any treatment prior to CNS involvement due to they initially presented with CNS lesions, and were diagnosed as SCNSL later.

**Table 1:** Clinical characteristics of SCNSL patients

Characteristics	N	%
Age at initial disease, mean (range)	59 (20–76)	
≤ 60	13	50.0
> 60	13	50.0
Gender		
Male	14	53.8
Female	12	46.2
Primary site		
Lymph node	12	46.2
Extranodal	14	53.8
Breast	4	15.4
Testicular	3	11.5
Others	7	26.9
CNS Relapse Type		
Isolated disease	21	80.8
CNS with disease progression	1	3.8
Combined disease	5	15.4
CNS relapse time, median (range)	3 (0–10)	
< 5 years	19	73.1
≥ 5 years	7	26.9
Performance Status at CNS relapse		
0–1	18	69.2
2–4	8	30.8
Diagnosis approaches		
Biopsy or surgery resection	24	92.3
Enhanced MRI	2	7.7

### Clinical and physiological findings, relapse site, pathological findings, and treatment at CNS relapse

All patients presented with brain parenchymal lesions, and one patient also had spinal cord compression. The symptoms of CNS relapse varied with location; the most common symptom was headache, and no epilepsy was observed in our study. Eye symptoms, such as blurred vision, were observed in 26.9% (*n* = 7) of the patients. The time from clinical presentation to a definite diagnosis ranged from 4 to 180 days (median 30 days). One patient died of post-operation intracranial hemorrhage. Three patients presented to our center initially as PCNSL but were later detected as having systemic disease and were distributed to SCNSL.

**Fig. 1:**
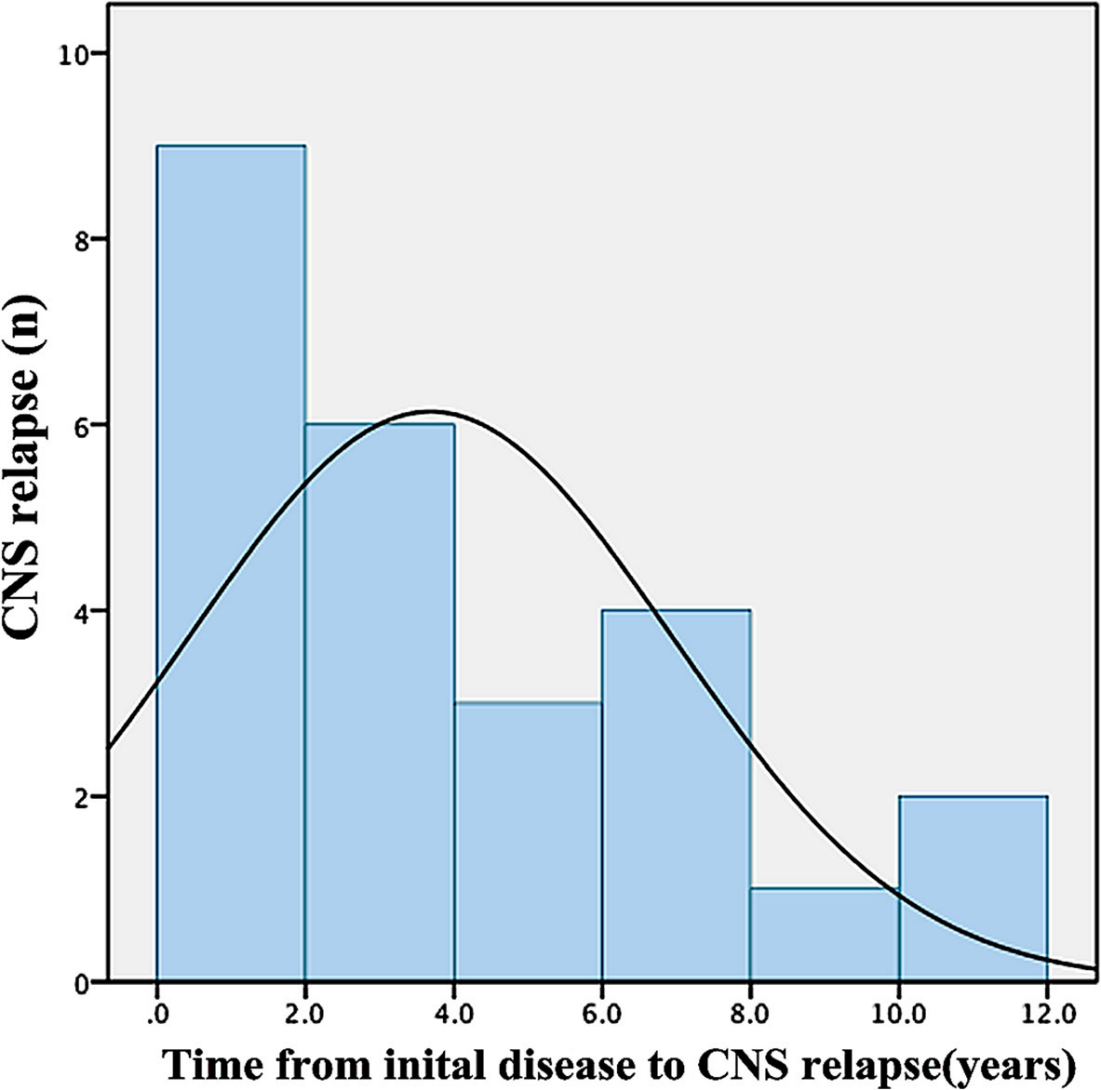
Distribution of relapse times from initial diagnosis of systemic disease in SCNSL patients

In this study, 80.8% (*n* = 21) of the patients were categorized as having isolated CNS relapse, 3.8% (*n* = 1) had CNS with disease progression, 15.4% (*n* = 4) had combined disease, and those in whom CNS involvement was found after the first year of systemic disease were more likely to have isolated CNS relapse (*p* = 0.034) (Table 2). Regarding the time of relapse, 73.1% (*n* = 19) had CNS relapse within the first five years after diagnosis with systemic disease with a median CNS relapse time of 3 years (Fig. 1). 88.5% (*n* = 23) patients underwent stereotactic biopsy, only 3.8% (*n* = 1) patient received intracranial tumor resection, and 77.0% (*n* = 20) were diagnosed with enhanced MRI. Pathological results showed that all were DLBCL, and of these, 92.3% (*n* = 24) were non-germinal center DLBCL subtypes, while others were germinal center B cell (GCB) subtype. BCL2 and BCL6 expression was detected in 69.2% (*n* = 18) of the patients, MYC was positive in 15 out of 16 (93.7%) of the SCNSL patients, and 93.8% presented with Ki-67 higher than 90%.

**Table 2:** Clinical characteristics and CNS relapse types

Characteristics	Isolated CNS	Synchronic CNS and systemic disease	*P* value*
	*N* (%)	*N* (%)	
Age			
≤ 60	10 (47.6)	3 (60.0)	1.000
> 60	11 (52.4)	2 (40.0)	
Relapse time			
≤ 1 year	5 (23.8)	4 (80.0)	0.034
> 1 year	16 (76.2)	1 (20.0)	
Primary site			
Lymph node	9 (42.9)	3 (60.0)	0.635
Extra node	12 (57.1)	2 (40.0)	
Intravenous MTX			
Yes	2 (9.5)	0 (0.0)	1.000
No	19 (90.5)	5 (100)	
Rituximab			
Yes	10 (47.6)	1 (20.0)	0.356
No	11 (52.4)	4 (80.0)	

Methotrexate, MTX; * Fisher’s Exact Test

### Clinical and physiological findings, pathological findings of PCNSL patients

All PCNSL patients had parenchymal diseases, their median age was 56.5 years (range 28-82 years). 96.2% (*n* = 25) patients underwent stereotactic biopsy, 3.8%(*n* = 1) patient was diagnosed with intracranial tumor resection. As for pathological findings, all were DLBCL, with 92.3% (*n* = 24) non-germinal center DLBCL subtypes, and 7.7%(n = 2) germinal center B cell (GCB) subtype. A detailed table of patients survival is shown in Additional file 1.

### MRI findings in SCNSL and PCNSL patients

All PCNSL patients avoided steroid treatment before MRI and surgery while 6 SCNSL patients with isolated CNS disease used corticosteroids before diagnosis.

*Multiplicity and localization* Parenchymal involvement was present in all SCNSL patients (Table 3), with multiple lesions found in 76.9% (*n* = 20) of the cases; in PCNSL, this proportion was 42.3% (*n* = 11) (*p* = 0.011). The SCNSL lesions were located in the deep gray matter in 69.2% (*n* = 18) and in the white matter in 80.8% (*n* = 21) of the patients; in PCNSL, these ratios were 46.2% (*n* = 12) and 65.4% (*n* = 17). Brainstem involvement was detected in only 11.5% (*n* = 3) of SCNSL cases but was observed in 34.6% (*n* = 9) of PCNSL patients (*p* = 0.100). In SCNSL, supratentorial lesions were seen in 65.4% (*n* = 17) of the cases and concomitant supratentorial and infratentorial lesions in 34.6% (*n* = 9), and none of them had solitary infratentorial lesions. Among the PCNSL patients, 23.1% (*n* = 6) had solitary infratentorial lesions (*p* = 0.003).

**Table 3:** Results of statistical analyses of radiological evaluation regarding location, enhancement pattern, multiplicity of the lesions between PCNSL and SCNSL

	PCNSL group *N* (%)	SCNSL group *N* (%)	*P* value
Gender			1.000
Male	14 (53.8)	14 (53.8)	
Female	12 (56.2)	12 (53.8)	
Age			0.402
≤ 60	16 (61.5)	13 (50.0)	
> 60	10 (38.5)	13 (50.0)	
Performance Status			0.026
0–1	10 (38.5)	18 (69.2)	
2–4	16 (61.5)	8 (30.8)	
Multiplicity			0.011
Single	15 (57.7)	6 (23.1)	
Multiple	11 (42.3)	20 (76.9)	
Butterfly pattern			1.000
Yes	2 (7.7)	3 (11.5)	
No	24 (92.3)	23 (88.5)	
T1W^a^			0.671
Hypo	21 (80.8)	19 (79.2)	
Iso	4 (15.4)	3 (12.5)	
Hypo-Iso	0 (0.0)	1 (4.2)	
Hyper	1 (3.8)	1 (4.2)	
T2W^b^			0.051
Hyper	24 (92.3)	16 (66.7)	
Iso	2 (7.7)	4 (16.7)	
Hyper-Iso	0 (0.0)	1 (4.2)	
Hypo	0 (0.0)	3 (12.5)	
T2 Flair^c^			0.253
Hyper	25 (96.2)	11 (84.6)	
Iso or Hypo	1 (3.8)	2 (15.4)	
DWI^d^			0.049
Hyper	26 (100)	13 (81.3)	
Non-hyper	0 (0.0)	3 (18.8)	
Enhancement			0.383
Homogeneous	16 (61.5)	16 (61.5)	
Patchy	8 (30.8)	6 (23.1)	
Ringlike	2 (7.7)	2 (7.7)	
No enhancement	0 (0.0)	2 (7.7)	
Location of Lesion (s)			
Deep grey matter	12 (46.2)	18 (69.2)	0.092
White matter	17 (65.4)	21 (80.8)	0.211
Cerebellum	6 (23.1)	5 (19.2)	0.734
Brainstem	9 (34.6)	3 (11.5)	0.100
Supra or Infra			0.003
Supratentorial	17 (65.4)	17 (65.4)	
Infratentorial	6 (23.1)	0 (0.0)	
Both	3 (11.5)	9 (34.6)	
Survival status^e^			0.291
Alive	23 (88.5)	19 (73.1)	
Death	3 (11.5)	7 (26.9)	

Diffusion weighted imaging, DWI; T2 fluid-attenuated inversion-recovery, T2 Flair; T1 weighted imaging, T1W; T2 weighted imaging, T2W;a、b:T1W and T2W were available in 24 SCNSL patients; c:T2 Flair imagines were done in 13 SCNSL patients; d: DWI imagines were done in 16 SCNSL patients; e: Details of survival seen in supplement materials

*Signal characteristics* The signal characteristics of SCNSL and PCNSL were quite similar. On T1-weighted (T1W) images, lesions were hypointense in 79.2% (*n* = 19), hyperintense in 4.2% (*n* = 1), and isointense in 12.5% (*n* = 3) of SCNSLs. The T2-weighted (T2W) signal of the lesions was hyperintense in 66.7% (*n* = 16) of SCNSL and 92.3% (*n* = 24) of PCNSL patients. T2 Flair hyperintensity was detected in 84.6% (*n* = 11) of the patients. Diffusion-weighted imaging (DWI) hyperintensity was found in 81.3% (*n* = 13) of the SCNSL patients, while all of the PCNSL patients presented with hyperintensity on DWI (*p* = 0.049).

**Fig. 2:**
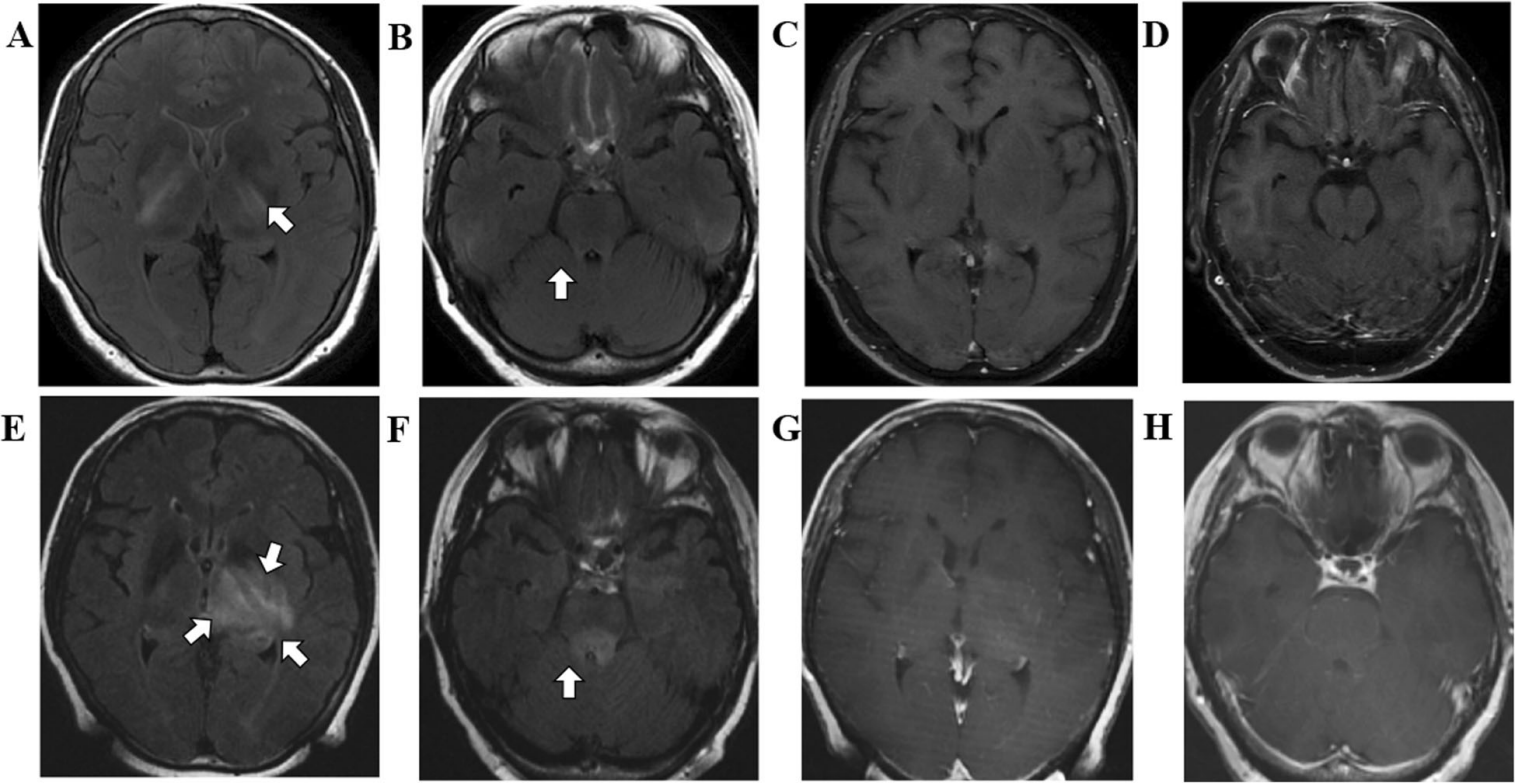
Multiple patchy hyperintensity on T2 Flair (**a** and **b**: arrows) were found in the right cerebellar hemisphere, bilateral cerebral peduncle, bilateral basal ganglia, thalamus, but enhancement was not observed (**c** and **d**). Re-examinations one month later via T2 Flair revealed some enlarged lesions without enhancement (**e** and **f**: arrows) and some still without enhancement (**g** and **h**), and stereotactic biopsy confirmed DLBCL

**Fig. 3:**
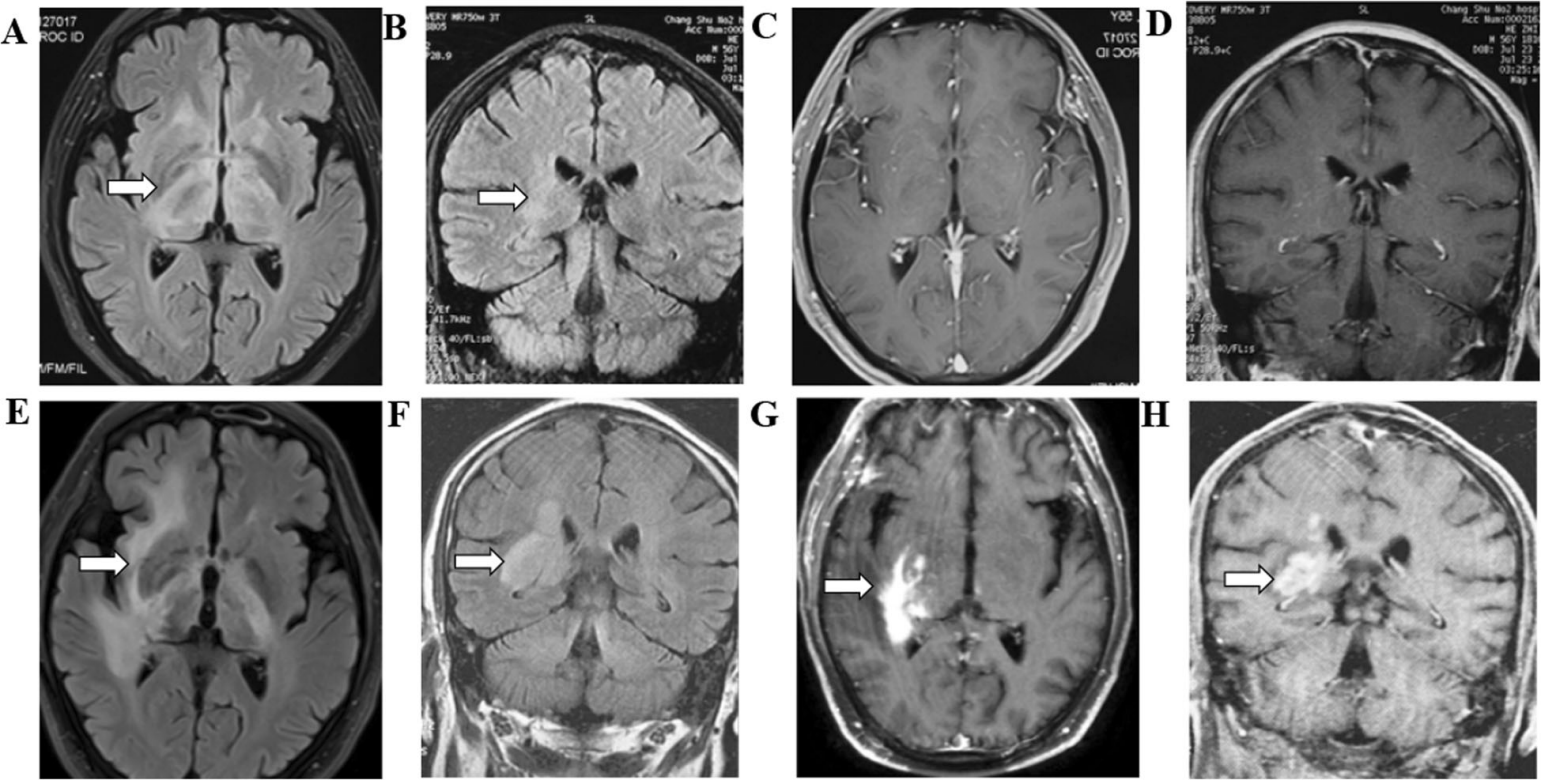
SCNSL without enhancement. Multiple lesions in the brain parenchyma showed hyperintensity on T2 Flair images (**a** and **b**; arrows). No enhancement was observed on MRI (**c** and **d**). Two months later, with the progression of the disease, the volume of lesions was observed on T2 Flair (**e** and **f**: arrows), strong enhancement was present after gadolinium injection in T1-weighted images (**g** and **h**: arrows), and stereotactic biopsy confirmed DLBCL CNS involvement

*Enhancement pattern* In the SCNSL group, the enhancement pattern was homogenous nodular in 61.5% (*n* = 16), patchy in 23.1% (*n* = 6) and ring-like in 7.7% (*n* = 2) of the cases. Notably, 7.7% (*n* = 2) of the patients presented with lesions without enhancement (Fig. 2). One SCNSL patient initially had no enhancement on MRI and was diagnosed with anti-NMDA-receptor encephalitis, but eventually, with the progression of the disease, the tumor developed enhancement, and stereotactic biopsy confirmed DLBCL with CNS involvement (Fig. 3**)**.

## Discussion

In the Rituximab era, the rate of CNS relapse of DLCBL in the form of parenchymal disease is increasing [15]. This condition accounts for high mortality [2] and shortened overall survival of less than 6 months [3]. Early diagnosis of CNS events is critical for successful treatment and improved prognosis. Some patients with typical MRI features, conventional cerebrospinal fluid (CSF) cytology and CSF flow cytometry tests could allow a definite diagnosis of SCNSL. However, in some patients, MRI features could be untypical at the initial of CNS relapse, making it difficult to confirm diagnosis. Stereotactic biopsy is a standard procedure in PCNSL diagnosis but is not routinely observed in SCNSL due to its invasion and its relatively limited sensitivity (20–65% in immunocompetent patients) [7, 8, 16]. In order to achieve early diagnosis of SCNSL and differential diagnosis before systemic evaluations, we made an comparison between MRI of SCNSL and PCNSL, and we find out some unique MRI patterns of SCNSL.

CNS relapse in DLBCL is reported to mainly occur within the first year after diagnosis (median, 6 months) [17]. In our study, the median CNS relapse time was 3 months, which corresponds to previous reports. The distribution of CNS involvement times also indicates that early relapse or concurrent disease is not rare in SCNSL groups, suggesting that affected patients harbor occult malignant cells in the CNS at diagnosis [18–20]. The incidence of CNS relapse decreased after the introduction of rituximab following a change in the pattern of CNS relapse, with a predominance of parenchymal over leptomeningeal relapse and of isolated over combined (systemic plus CNS) relapse [2]. Increasing reports indicated that SCNSL presents as a parenchymal disease [19, 21–23]. Hana Malikova et al. recently presented a series of SCNSL cases in which parenchymal lesions occurred in 18 out of 21 cases, indicating that SCNSL presents as a parenchymal disease.

Efsun Senocak reported that SCNSL predominantly presents as multiple lesions, while deep gray matter and infratentorial involvement were scarce comparing with PCNSL but not statistically significant [24]. In our study, SCNSL presented with multiple lesions, in contrast to PCNSL (*p* = 0.011), and infratentorial and brainstem involvement were significantly rarer in SCNSL patients. In Senocak’s report, lesions were multiple in 58.3%(*n* = 7) PCNSL, whereas, this number was smaller (42.3%(*n* = 11)) in our research, still we came to an conclusion that SCNSL predominantly presents as multiple lesions. The difference between ours and previous study may due to larger sample size, though our study indicated there was a statistically significant difference in the investigated MR features between two groups, they had similar trends.

The lesions of two SCNSL patients were discovered by examining the hyperintensity on T2 Flair as there was no enhancement shown. However, the mechanism remains unknown. In addition, according to Tabouret et al., in PCNSL, as nonenhancing Flair abnormalities may exacerbate the overall tumor burden, T2-weighted/Flair sequences should also be taken into consideration [25]. On the other hand, for the above two patients, we postulate that this pattern of nonenhanced lymphoma may be due to the alternated immune state brought by corticosteroid-containing chemotherapy, as both of them have received the standard treatment for systemic DLBCL when the CNS lesions occurred. Koubska et al. found that there were statistically significant differences in morphological MRI findings between immunocompromised and immunocompetent patients with CNSL. The authors speculated that the difference in enhancement pattern between immunocompromised and immunocompetent patients may be correlated to corticosteroid therapy [26]. Hana Malikova et al. also introduced varied MR performance in SCNSL in their study, which showed that SCNSL can mimic progressive multifocal leukoencephalopathy and multiple ischemic lesions.

Moreover, this could be a unique manifestation of SCNSL, and further research should explore the correlation between MRI features and biological characteristics.

## Conclusions

Due to the rarity of parenchymal SCNSL, very few studies have summarized its characteristics on MRI. This study provides an overlook of the characteristics of both clinical and MRI presentations in SCNSL patients. Additionally, we compared SCNSL and PCNSL to further identify their unique radiological findings. The majority of parenchymal involvement occurred within the first year of systemic lymphoma, and those in whom CNS involvement was found after the first year of systemic disease were more likely to have isolated CNS relapse. For MRI features, SCNSL mostly presented at multiple and supratentorial locations and was significantly different from PCNSL in this regard. Moreover, nonenhancement MRI could not rule out the possibility of SCNSL, T2 Flair may provide more information, and dynamic monitoring on MRI could help in patient diagnosis.

## Supplementary information

**Additional file 1.** Living status of SCNSL and PCNSL patients. This data described the living status of patients referred in this article by the initial of data analysis.

## Data Availability

The datasets supporting the conclusions of this study are available from the corresponding author on reasonable request.

## Abbreviations

CNS: Central nervous system
CSF: Cerebrospinal fluid
DWI: Diffusion weighted imaging
Flair: Fluid-attenuated inversion recovery
HD-MTX: High-dose methotrexate
PCNSL: Primary central nervous system lymphoma
PFS: Progression free survival
SCNSL: Secondary central nervous system lymphoma
T2W: T2-weighted imaging
WBRT: Whole brain radiotherapy
